# Pain acceptance and personal control in pain relief in two maternity care models: a cross-national comparison of Belgium and the Netherlands

**DOI:** 10.1186/1472-6963-10-268

**Published:** 2010-09-10

**Authors:** Wendy Christiaens, Mieke Verhaeghe, Piet Bracke

**Affiliations:** 1 Department of Sociology, Ghent University, Ghent, Belgium

## Abstract

**Background:**

A cross-national comparison of Belgian and Dutch childbearing women allows us to gain insight into the relative importance of pain acceptance and personal control in pain relief in 2 maternity care models. Although Belgium and the Netherlands are neighbouring countries sharing the same language, political system and geography, they are characterised by a different organisation of health care, particularly in maternity care. In Belgium the medical risks of childbirth are emphasised but neutralised by a strong belief in the merits of the medical model. Labour pain is perceived as a needless inconvenience easily resolved by means of pain medication. In the Netherlands the midwifery model of care defines childbirth as a normal physiological process and family event. Labour pain is perceived as an ally in the birth process.

**Methods:**

Women were invited to participate in the study by independent midwives and obstetricians during antenatal visits in 2004-2005. Two questionnaires were filled out by 611 women, one at 30 weeks of pregnancy and one within the first 2 weeks after childbirth either at home or in a hospital. However, only women having a hospital birth without obstetric intervention (N = 327) were included in this analysis. A logistic regression analysis has been performed.

**Results:**

Labour pain acceptance and personal control in pain relief render pain medication use during labour less likely, especially if they occur together. Apart from this general result, we also find large country differences. Dutch women with a normal hospital birth are six times less likely to use pain medication during labour, compared to their Belgian counterparts. This country difference cannot be explained by labour pain acceptance, since - in contrast to our working hypothesis - Dutch and Belgian women giving birth in a hospital setting are characterised by a similar labour pain acceptance. Our findings suggest that personal control in pain relief can partially explain the country differences in coping with labour pain. For Dutch women we find that the use of pain medication is lowest if women experience control over the reception of pain medication and have a positive attitude towards labour pain. In Belgium however, not personal control over the use of pain relief predicts the use of pain medication, but negative attitudes towards labour.

**Conclusions:**

Apart from individual level determinants, such as length of labour or pain acceptance, our findings suggest that the maternity care context is of major importance in the study of the management of labour pain. The pain medication use in Belgian hospital maternity care is high and is very sensitive to negative attitudes towards labour pain. In the Netherlands, on the contrary, pain medication use is already low. This can partially be explained by a low degree of personal control in pain relief, especially when co-occurring with positive pain attitudes.

## Background

Women are increasingly encouraged to take an active role in decision-making regarding pregnancy, labour and delivery [1,2]. As a consequence of women's increased involvement, their attitudes and beliefs have become a new domain of interest. In contrast to other medical interventions in the perinatal period, the use of pain relief is left mainly to a woman's choice [3]. The use of labour analgesia is mostly researched as an independent variable to assess its effects on maternal health and wellbeing, e.g., maternal satisfaction [4]. Some studies have investigated the reasons for pain relief during labour. Demographic and personality characteristics of the mother [5], clinical, structural and organisation factors [6-8], patient and caregiver preferences [8-10], beliefs about childbirth and labour pain [10-12] and perceived and preferred control over the childbirth situation [10] have been shown to influence the use of pain relief. Other antecedents to the use of pain relief are the intention/preference to use pain relief [13,14], pain expectation [1,15], knowledge about labour analgesia [16] and antenatal classes [17].

The reaction to labour pain has been studied among women with different cultural backgrounds. Examples are the studies of Senden et al. [18], comparing parturients in a Dutch and American hospital, and Weisenberg and Caspi [19], testing the influence of cultural group of origin on the reaction to childbirth pain. Also variation in labour pain experiences between several birth settings (e.g. home and hospital) has been researched [20]. We will specifically address the relative impact of antenatal pain acceptance and personal control in pain relief on the use of pharmacologic pain relief during labour and delivery in Dutch and Belgian hospital contexts. The aim of this paper is twofold. First, we want to address the predictive value of labour pain acceptance and personal control in pain relief for the use of pain medication during childbirth (See RQ1). Second, we will introduce care context by the comparison of the Belgian and Dutch maternity care systems (See RQ2 and RQ3).

### Pain acceptance and personal control in pain relief

The first research question (RQ1) addressed in this paper is whether acceptance of labour pain and personal control in pain relief determine the way childbearing women cope with labour pain. Pain acceptance, or the willingness to experience pain [21], has emerged as an important condition that reduces the suffering that often accompanies the experience of pain [22-25]. For example Waldenstrom et al. [26] reported that women with negative pain attitudes experienced more pain and were more anxious during labour. Fear is commonly found to be associated with increased labour pain [27-30]. The non-acceptance of pain is associated with the need for pain reduction, while acceptance results in lower emotional distress [25]. Heinze and Sleigh [11] found that women who laboured with an epidural had a lot of fear about childbirth, an external locus of control for childbirth, and a desire to remain passive in the childbirth process. Positive pain attitudes or confidence, as opposed to fear, have been shown to decrease pain perception and pain medication use [31,32]. In line with these findings, it is our hypothesis that the acceptance of labour pain will result in less need for pain medication during childbirth.

In general, personal control is one of the main determinants of maternal satisfaction with childbirth [33-40] When narrowed down to labour pain, personal control is about women's active role in the decision to have or refrain from having pain relief during labour [41]. Based on women's perceptions of control as described in the literature and on their own experience in midwifery, McCrea and Wright [4] define personal control as 'a) the women's feeling of being in control as opposed to staff being in control; b) their input into decision-making governing pain medication; and c) use of personal coping resources to cope with labour pain'. Only a few investigations have been done with regard to personal control in pain relief. In addition to the psycho-social determinants of personal control in pain relief [42], its impact on satisfaction with pain relief during labour has been investigated [4]. However, our research question--is personal control in pain relief associated with pain medication use during childbirth--has not yet been addressed. We expect that personal control in pain relief as such will not be predictive of pain medication use, but will interact with pain acceptance. It will reduce pain medication use in women with positive pain attitudes and increase it in women with negative attitudes towards labour pain. This hypothesis is in accordance with Heinze and Sleigh's argument [11] that women's preferences and beliefs may have more influence on the management of labour pain than situational factors, such as personal control in pain relief. In fact, we assume that personal control in pain relief will be bound more by context than pain acceptance since it is dependent on what the hospital staff allows for.

### The role of the care context

**Figure 1:**
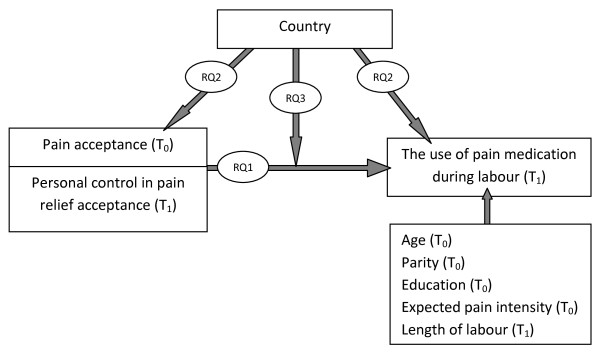
**Causal diagram**. RQ: Research Question. T0: Time zero, 30 weeks of pregnancy. T1: Time 1, within 2 weeks after birth

The second and third research questions concern the role of the care context. While it is important to recognise individual characteristics (e.g., pain acceptance and personal control) when explaining the use of pain medication, it is equally important to consider the interplay of these factors with the social contexts in which pain medication is used [43]. As our second research question (RQ2) we want to assess the contribution of the Belgian and Dutch care context to 1) the pain acceptance and personal control in pain relief and 2) the medication use during labour. In a third step (RQ3), a cross-national comparison of Belgian and Dutch childbearing women allows us to gain insight into the relative importance of pain acceptance and personal control in pain relief in two maternity care models. The three research questions and variables included in this investigation are represented in figure 1.

Although they are neighbouring countries sharing the same language, political system and geography, Belgium and the Netherlands are characterised by a different organisation of health care, particularly in maternity care. In Belgium the medical risks of childbirth are emphasised but neutralised by a strong belief in the merits of the medical model [44,45]. In line with the midwifery model of care, in the Netherlands childbirth is defined as a normal physiological process and family event [46].

These different approaches to childbirth are reflected in the organisation and utilisation of maternity care facilities. In the Netherlands, for example, home births are encouraged by directing women expecting a normal birth into primary care [47], resulting in a 21.5% home birth rate [48]. The option of a policlinical birth, or a 'home birth away from home', (11.3%) [48] provides women with the possibility of having a midwife-led hospital birth with a short stay after the baby is born [49]. In case of difficulties during pregnancy and labour, women are referred to specialist care [50]. The relatively high proportion of home births and the emphasis on normality result in low rates of obstetric interventions [51]. In contrast, in Belgium 97.9% of childbearing women prefer to have their babies in hospital, finding reassurance in the proximity of obstetric technology. Home births are most popular in Flanders with 1.4%, compared to Brussels with 0.4% and Wallonia with 0.3% [52]. Not surprisingly, Belgium has been characterised by higher obstetric intervention rates compared to the Netherlands. However, over time the obstetric intervention rates of both countries have been converging and even reversing. For example in 2003 the Flemish induction rate was 30% and the percentage of caesarean sections was 18.3% [53], versus 28.8% and 14.8%, respectively, in the Netherlands [54]. In 2007 caesarean section rates were 19% in Flanders and 15.4% in the Netherlands. In that year Dutch induction rates (33%) however, exceeded the Belgian induction rates (25.6%). The Flemish figures are comparable to the numbers in Brussels, where the section rate was 16.6% and the induction rate 27.8% in 2004 [55]. So far, these data are unavailable for Wallonia. The difference is especially large regarding the use of pain medication during labour. The Dutch organisation for perinatal epidemiology (SPRN) reports that the Netherlands stands out for its low use of pain relief during labour. In 2007, in 9.6% of all Dutch deliveries, an epidural had been administered [56]. In Belgium the use of epidural analgesia has doubled since 1991, from 32% to 66.6% in 2007[48,57].

Some authors found indications of a specific pain culture in the Netherlands. Dutch women showed a greater acceptance of labour pain compared to Americans in Senden's study [18]: nearly two thirds of the Dutch women laboured without pain medication, compared to one-sixth of the Americans. Jordan [58] concluded from a comparison of childbearing women in Yucatan, Sweden, the Netherlands and the United States that the majority of Dutch women do not expect or receive pain medication during labour. The distinctive Dutch ideas about pain and discomfort, which are reflected in a low use of pain medication, is also mentioned by DeVries [46]: "Dutch ideas about pain and the value of medication (...) are reflected in their relatively low use of pain and other medications compared to other nations in the European Union." (p. 158). Belgian maternity care is characterised by the medical model in which pain is viewed as controllable and needless [59]. Consequently, we tend to believe that Belgian and Dutch women are socialised in different pain cultures as part of the dominant models of maternity care. In the women-centred approach, conform the mastery model [60] pain acceptance and personal control are believed to be valuable coping strategies [42]. Pain is perceived as an ally in the birth process. Pain serves a biological purpose and is seen as constructive [61-63]. In the pain management model [60] however pain is perceived as a needless inconvenience easily resolved by means of pain medication [59]. In addition, in the biomedical care ideology, personal control may conflict with the control of the staff health professionals. They might experience women's personal control as encroaching on their expertise and on their decision-making role. In conformity with this biomedical ideology and practice, we assume that Belgian women planning a hospital birth are more likely to reject labour pain, to try to avoid it and to rely on health professionals to control it. Therefore we expect them to score low on pain acceptance and low on personal control in pain relief. In the Netherlands we think the opposite is more likely because the midwifery model, which we assume also permeates into Dutch hospitals, defends the view that labour pain serves a biological purpose and that relieving it might disturb the natural birth process [64].

## Methods

### Selection of method

In a cross-national comparative investigation of Belgian and Dutch childbearing women we assessed how the organisation of maternity care was related to antenatal and postnatal outcomes, such as satisfaction with childbirth [39,45,65] and childbirth expectations and experiences [66]. In this paper we focus on the use of pain medication during labour. In order to be able to quantify the contribution of the maternity care system to women's childbirth experiences and to reach as many women as possible in a short period of time, two questionnaire surveys were carried out: one at 30 weeks of pregnancy and one within two weeks after childbirth. A 2 weeks postpartum time frame was chosen to minimise the effect of inaccurate recall on reports of the birth experience, following the approach used by Ayers and Pickering [67]. Women were invited to participate over a 3-month period. Five to 8 months elapsed from invitation to participate to completion of the last questionnaire. As data collection was not carried out simultaneously in each hospital or midwifery practice, 1 year was necessary to gather all the data (September 2004 - September 2005).

### Measurement

#### Dependent variable

Use of pain medication was the dichotomous dependent variable on which the logistic regression was performed. It was assessed by asking our respondents the following question: 'Did you use pain medication during labour?' (no = 0; yes = 1). In 90% of the cases the pain medication used was epidural analgesia.

#### Independent variables

To measure pain acceptance pregnant women were asked to indicate to what extent they agreed with the following two statements: 'I desire to deliver without epidural analgesia' and 'Pain is needless'. Answers were scored on a 5-point Likert scale ranging from 'strongly agree' to 'strongly disagree'. Both items were coded in the same direction and merged into one scale by averaging the scores on both items. High values represent pain acceptance or positive pain attitudes. The Cronbach's alpha of the acceptance scale was 0.66, which is acceptable especially for a scale of two items [68].

Personal control in pain relief was measured by the Personal Control in Pain Relief Scale, designed by McCrea and Wright [4]. It consists of a modified version of Slade et al.'s [69] personal control scale and contains eight items. McCrea and Wright used visual analogue scales and women were asked to put a cross on a 10 cm line at the point that best described their perception of the control they had over pain relief. Each item was anchored with opposing answer categories, for example 'Could not control it at all' versus 'could control it completely'. We added numbers from one through ten to the 10 cm line. Examples of items are 'Who was most in control of the way your labour pain was managed?' 'How much were you able to control the pain you felt during labour?' and 'How much were the midwives/doctors able to control the pain you felt during labour?' Two items were not taken into account in this analysis. They concerned the use of exercises learned at antenatal classes and were therefore of less relevance. Internal consistency was satisfactory, with a Cronbach's alpha of 0.69. Both pain acceptance and personal control in pain relief were centred around the grand mean.

#### Control variables

Two Visual Analogue Scales (VAS)--one about labour and one about delivery--were used to measure the expected intensity of labour pain, ranging from 'no pain at all' (0) to 'unbearable pain' (100). Respondents were asked, 'How much pain do you expect to feel during labour?' and 'How much pain do you expect to feel during delivery?' Cronbach's alfa was 0.53, which is low but acceptable for a two item scale [68]. Mean scores were calculated to merge both questions into one indicator of the pain intensity. The measurement of labour pain by visual analogue scales is common practice in research on childbirth [70-72] and has been found to be reliable. Compared to more complex pain measures, the VAS is preferable [73,74].

The length of labour has been calculated by means of two questions: "When did contractions begin?" and "When was your baby born?" Both date and hour were filled in by the respondents. The measurement unit of the difference between these two time points was initially minutes, but has been transformed into hours in order to make the odds ratios more meaningful and easier to interpret.

We also took into account a number of personal characteristics of the childbearing women, such as parity (0 = primiparous; 1 = multiparous), age in years (centred around grand mean) and educational level (0 = no higher education; 1 = higher education).

### Population and sample

The study concerns two comparable cities in the Belgian and Dutch regions, Ghent and Tilburg, respectively. Although we do not claim representativeness, we will refer to Belgium and the Netherlands, and the Belgian and the Dutch to enhance the readability of this paper. Since the total population of pregnant women could not be determined, we had to rely on a convenience sample. In Ghent there are four hospitals of which three agreed to participate. We have no reason to believe that the population of the missing hospital differs from the populations of the participating hospitals. In Tilburg both hospitals agreed to cooperate. At each hospital pain relief, more specifically epidural analgesia, was available on a 24-hour basis.

In addition, we contacted six midwifery practices in Tilburg to reach enough women planning a home birth. In Belgium, Ghent does not have enough midwifery practices to attain the same number of home births. Therefore, we went beyond the city borders of Ghent and contacted 21 midwifery practices across Flanders. Although women who had a home birth were excluded from our analyses, we want to emphasise that they were surveyed as part of the bigger project. We also ask the attention of the reader for the fact that women planning for a home birth, but who were referred to the hospital between their thirtieth week of pregnancy and the moment of birth (including labour), were included in our analysis. For Belgium this is the case for 16 respondents, for the Netherlands 89. This difference reflects the large number of referrals in the Netherlands (see e.g. [50]).

Sample size calculations based on a 0.95 confidence interval suggested we needed 600 study participants for a reliable statistical analysis. At 30 weeks of pregnancy, 827 women filled out the antenatal questionnaire; 611 of those women also participated in the study during the first 2 weeks after delivery and completed the second questionnaire.

Since we needed information about both time points for our analysis, our initial sample counted 611 respondents. After exclusion of home births (n = 179), Caesarean sections (n = 84) and the cases with missings on the variables educational level (n = 13), pain relief (n = 5) and place of delivery (n = 3), a working sample of 327 childbearing women consisting of 157 Belgian and 170 Dutch women remained. Because pharmacological pain relief is not available at home, only spontaneous vaginal deliveries in hospital settings were included. We excluded women with obstetric interventions, such as caesarean section or forceps delivery, because it was thought that in the case of an obstetric intervention women would not have been involved actively in decision-making regarding the use of pain relief. Also, the acceptance of labour pain seems irrelevant in such a situation.

### Procedure

During prenatal visits, women were asked by their midwife or obstetrician to participate in the research project, in order to include both home and hospital births. Inclusion criteria were broad: both Belgian and Dutch women had to speak and understand Dutch and had to be 18 years or older. The antenatal questionnaire was handed out during an antenatal visit at 30 weeks of pregnancy together with an information sheet. It was returned to the obstetrician or midwife during one of the following antenatal visits. Within a few days after delivery, women received the postnatal questionnaire either from the medical staff in the case of a hospital birth, or from the midwife in the case of a home birth. Women who delivered in a hospital completed the postnatal questionnaire during their postpartum stay in the maternity ward. Women with a short stay or home birth, however, responded by direct mail instead. Antenatal and postnatal questionnaires were given a code to facilitate the merging of the antenatal and postnatal information from each respondent.

Women were recruited during prenatal visits to their obstetricians and midwives. Therefore, we had little control over the inclusion process and, consequentially, the response rate. Although we asked that women who refused to participate be registered, this was not systematically done by every hospital. As a result, we do not know the exact number of women invited to participate in this study. To calculate the response rate we used the number of provided questionnaires; that number is based on an estimate of eligible women made by midwives and obstetricians acting as proxy. The response rate was calculated by dividing the number of respondents by the number of provided questionnaires. This calculation resulted in an average response rate of 43% (n = 238) for all Belgian hospitals, 41% (n = 137) for Belgian midwifery practices, 42% (n = 208) for Dutch hospitals and 54% (n = 244) for Dutch midwifery practices. For hospitals the smallest response rate was 19%, the highest 68%. For the midwifery practices the response rate was 38% and 100%, respectively. However, we know that not all questionnaires were distributed, which means that our estimations of the response rates are in fact very conservative.

### Ethical considerations

A written informed consent was required of all respondents. Anonymity was guaranteed, since the researchers have no information about the identity of the respondent. The Committee for Ethics of the University Hospital has approved the study. Ethical approval was gained in Ghent only. In the Netherlands, approval from a research Ethics committee is not required if no interventions take place during the research. It was explained to potential participants that they were free to participate and that their privacy was guaranteed.

### Data analysis

After an exploration of the descriptives, a logistic regression analysis was performed using SPSS 15. The predictive value of the acceptance of labour pain and personal control in pain relief upon the actual use of pain relief was investigated. A logistic regression model has been constructed and the adjusted odds ratios (OR) calculated.

## Results

### Descriptives

Table 1 shows that the age of participating women ranged from 19 to 44 years with a mean age of 31.2 years; 30 for Belgian women and 32 for Dutch women. Those having their first baby made up 55.7% of all respondents; in Belgium 50.0% (n = 68) were having their first baby, in the Netherlands, 60.9% (n = 62). More Belgian (71.9%; n = 97) than Dutch (45.9%; n = 68) women completed higher education. Belgian women reported longer labours, with an average of almost 10 hours, compared to the Dutch with an average of 8.5 hours (t = 2.14, p = 0.03) (Table 1).

**Table 1:** Descriptive statistics

	Country	Mean	% (n)	SD	t or chi^2^	*p*
Higher education	Belgium	-	71.9 (97)	-	19.48	< 0.001
	the Netherlands	-	45.9 (68)	-		

Multiparae	Belgium	-	50.0 (68)	-	3.46	0.074
	the Netherlands	-	60.9 (62)	-		

Length of labour	Belgium	9.85	-	6.36	2.140	0.033
	the Netherlands	8.63	-	6.61		

Expected pain intensity	Belgium	63.76	-	18.08	0.96	0.338
	the Netherlands	61.84	-	15.72		

Age	Belgium	30.0	-	4.04	-4.66	<0.001
	the Netherlands	32.3	-	4.35		

Pain medication use	Belgium	-	47.8 (65)	-	37.80	<0.001
	the Netherlands	-	14.5 (22)	-		

Pain acceptance	Belgium	3.72	-	0.92	-0.39	0.694
	the Netherlands	3.75	-	0.78		

Personal control in pain relief	Belgium	7.07	-	1.39	7.95	<0.001
	the Netherlands	5.54	-	1.79		

Among Belgian women, 47.8% (n = 65) made use of pharmacological pain relief during labour or delivery, compared to 14.5% (n = 22) of the Dutch respondents. In both countries primiparous women are almost twice as likely to receive pain relief than multiparous women (Belgium: 57.9% versus 30.%; the Netherlands: 31.2% versus 17.3%). Dutch women expect about the same level of labour pain (mean = 61.84) as Belgian women (mean = 63.76). Dutch and Belgian women show the same average acceptance of labour pain (B: mean = 3.72; Nl: mean = 3.75; t = -.39; p = 0.694), but the Belgians report higher average scores on personal control in pain relief than the Dutch (B: mean = 7.07; Nl: mean = 5.54; t = 7.95; p < 0.001) (Table 1). Parity, length of labour and educational level especially may confound the comparison between Belgium and the Netherlands. Therefore these variables together with expected pain intensity and age were controlled for in the logistic regression model.

### Logistic regression model

Tables 2 shows the odds ratios and confidence intervals (CI) for the logistic regression models corresponding to the first and second research question, table 3 presents the same logistic regression model ran for Dutch and Belgian women separately, in order to answer the third research question.

**Table 2:** Logistic regression models with individual and country level predictors of pain medication use^1 ^(N = 327)

	Model 1				Model 2				Model 3			
	**OR**	**95% - CI** **lower - upper**		**p**	**OR**	**95% - CI** **lower - upper**		**p**	**OR**	**95% - CI** **lower - upper**		**p**

Intercept	0.289			<0.001	1.251			0.516	1.318			0.480

Age	0.912	0.851	0.977	**0.009**	0.980	0.913	1.053	0.590	0.983	0.910	1.062	0.658
Multiparous	0.833	0.451	1.539	0.560	0.887	0.480	1.640	0.703	0.735	0.376	1.437	0.368
Expected pain intensity	1.006	0.989	1.024	0.477	0.998	0.981	1.014	0.786	1.000	0.981	1.020	0.974
Highly educated	1.647	0.924	2.933	0.090	0.870	0.474	1.597	0.653	0.834	0.423	1.642	0.599
Length of labour	1.115	1.065	1.167	**<0.001**	1.123	1.073	1.176	**<0.001**	1.129	1.074	1.188	**<0.001**

Pain acceptance (a)	0.439	0.305	0.634	**<0.001**	-	-	-	-	0.435	0.292	0.647	**<0.001**
Personal control in pain relief (b)	0.991	0.834	1.117	0.915	-	-	-	-	0.721	0.583	0.892	**0.003**
(a)*(b)	0.613	0.485	0.776	**<0.001**	-	-	-	-	0.602	0.468	0.775	**<0.001**

Country (NL = 1, BE = 0)	-	-	-	-	0.134	0.071	0.252	**<0.001**	0.085	0.038	0.190	**<0.001**

**Table 3:** Logistic regression models with individual level predictors of pain medication use^1 ^for the Netherlands and Belgium separately

	the Netherlands				Belgium			
	**OR**	**95% - CI** **lower - upper**		**p**	**OR**	**95% - CI** **lower - upper**		**p**

Intercept	0.105			<0.001	1.255			0.665

Age	0.987	0.884	1.102	0.819	0.982	0.878	1.099	0.752
Multiparous	0.756	0.272	2.100	0.592	0.680	0.262	1.766	0.428
Expected pain intensity	1.012	0.979	1.046	0.493	0.995	0.969	1.022	0.725
Highly educated	1.019	0.371	2.797	0.971	0.705	0.264	1.883	0.486
Length of labour	1.088	1.019	1.163	**0.012**	1.181	1.089	1.281	**<0.001**

Pain acceptance (a)	0.792	0.356	1.763	0.568	0.260	0.138	0.487	**<0.001**
Personal control in pain relief (b)	0.642	0.460	0.895	**0.009**	0.845	0.633	1.129	0.254
(a)*(b)	0.660	0.449	0.970	**0.034**	0.684	0.427	1.096	0.114

In model 1 (table 2) the impact of labour pain acceptance and personal control on labour pain medication use is addressed (RQ1). What concerns the control variables, we find that longer labours (OR = 1.115 [1.065,1.167]) and younger age (OR = 0.912 [0.851,0.997]) rendered pain relief more likely. Expected pain intensity, level of education, and parity did not reach the 95% significance level. In line with our hypothesis, the interaction term 'pain acceptance*personal control' indicates that the likelihood of pain medication use is smallest if women have positive pain attitudes during pregnancy and report high personal control in pain relief after birth (OR = 0.613 [0.485,0.776]). In addition, the OR's of personal control in pain relief reveal that personal control in pain relief has no influence if women have average pain attitudes. Moreover, pain acceptance is the most important determinant of pain medication use during birth (OR = 0.439 [0.305,0.634]. This is also shown in the main effects model (no table) including only pain acceptance and personal control in labour pain, in addition to the control variables. In this main effect model only pain acceptance has a significant influence on pain medication use (pain acceptance: OR = 0.444[0.311,0.634]; personal control: OR = 1.187[0.997-1.413]).

In model 2 and 3 (table 2), conform the second research question, care context is introduced by adding the country variable to the analysis. First of all, we find that the use of labour pain medication is more likely among Belgian women (OR _model 2 _= 0.134 [0.071,0.252]; OR_model 3 _= 0.085 [0.038,0.190]). Secondly, in model 3 (table 2) it is shown that pain acceptance (OR = 0.435 [0.292,0.647]) and personal control in pain relief (OR = 0.721 [0.583-0.892]) reduce the likelihood of pain medication use, especially when they occur together (OR = 0.602 [0.468,0.775]. Thus, personal control in pain relief becomes a significant determinant of pain medication use, once the care context is introduced. This means that the country difference in pain medication use can be partially explained by differences in personal control in pain relief. We know from the descriptives that Dutch and Belgian women reported the same level of pain acceptance, while Belgians scored significantly higher than the Dutch on personal control in pain relief. This finding becomes more explicit in the results of the regression analyses for Belgium and the Netherlands separately.

In table 3, a similar regression model has been estimated for Belgian and Dutch women separately in order to answer the third research question: does the relative impact of labour pain acceptance and personal control in pain relief diverge between the Belgian and Dutch care context?

In Belgium, the likelihood of using pain relief is seriously reduced for women accepting labour pain (OR = 0.260 [0.138,0.487]). Personal control in pain relief, on the contrary, is of little importance (OR = 0.845 [0.633,1.129])). Also the co-occurrence of pain acceptance and personal control (OR = 0.684 [0.427,1,096] has no additional value.

For Dutch women a different picture arises from our results. The main determinant of pain relief shifts from labour pain acceptance towards personal control in pain relief. In table 3 two differences are important when comparing the country specific findings. First, for the Dutch women, the interaction term 'pain acceptance*personal control in pain relief' is significant. Second, for Dutch women not pain acceptance but personal control in pain relief is important in predicting pain medication use. This means that for Dutch women, especially personal control in pain relief (OR = 0.642 [0.460,0.895] has a significant reducing effect on medication use, even more so when co-occurring with pain acceptance (OR = 0.660 [0.449,0.970]).

**Figure 2:**
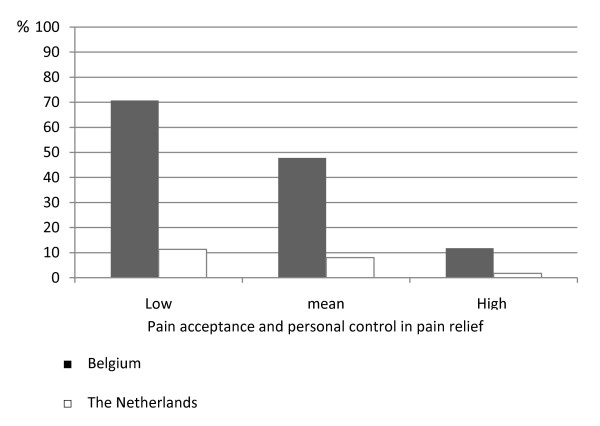
**Predicted likelihood of pain medication use for Belgium and the Netherlands**.

In Figure 2 we show the predicted likelihood of labour pain medication use estimated with the country specific model in table 3. This graph illustrates that, among the women who report low pain acceptance and personal control in pain relief (i.e., mean - 1SD), Belgians have a 71% chance of having their labour pain relieved, versus a likelihood of 11% for the Dutch. This could be an indication of an under-met need for pain relief on the part of the Dutch women with negative pain attitudes and little control over medication use. For the group with high labour pain acceptance and a lot of control over medication use (i.e., mean + 1SD), Belgian and Dutch women's chances of receiving pain medication are 12% and 2%, respectively. Thus, on both ends of the continua (pain acceptance and control over pain relief), Dutch women are about six times less likely than Belgians to receive pain medication. Belgian women accepting labour pain (with a normal vaginal birth) and controlling pain medication use, still have a 12% chance to get pain medication, which could indicate an over-met need.

## Discussion

In this study we investigated whether labour pain acceptance and personal control in pain relief influence the likelihood of pain medication use during childbirth. In addition we examined country differences in pain mediation use and especially its determinants among Belgian and Dutch childbearing women.

Before discussing the findings, we want to briefly list some of the shortcomings and merits of the study. First, our dataset is the result of a small convenience sample of childbearing women in two comparable Belgian and Dutch cities. This makes generalisability to the Belgian and the Dutch population uncertain, especially for the Netherlands because there is Dutch evidence of regional differences in birth outcomes and care facilities [75,76]. In addition, from comparison with national statistics (labour pain medication use in Belgium = 66.6%, in the Netherlands = 9.6%) [48,57], it is clear that in our sample Belgian users (47.8%) of pain medication are underrepresented, while Dutch users (14.5%) are overrepresented. This means that our estimations of country differences are rather conservative: in the population the Belgian and Dutch differences in the use of pain relief can be expected to be more pronounced. Also there were variations is response rates between hospitals. We have no reason to assume between hospital differences regarding the variables in our model. Although it is impossible to estimate the potential selection caused by the variation in response rates, two thoughts might be useful: 1) pain medication use (our dependent) was an unknown at the time of the invitation to participate in our study. Hence selection cannot affect this variable, 2) if there is a selection, we assume, it will be higher educated women being more willing to participate and easier to approach by the care provider. Higher educated women use more pain medication than lower educated women. This can result in an overestimation of the mean pain medication use. By consequence the selection of respondents by the care providers is a matter of concern. We agreed with the care providers that they would invite all women over the age of eighteen, understanding Dutch, and being in their 30th week of pregnancy, over a period of three months. We also emphasised that it was important to invite all women meeting these criteria, to avoid selection biases. We have no guarantees that this instruction has been followed.

Second, apart from sampling problems, some measurement issues should be mentioned. Scales of only two items were used to measure labour pain acceptance and labour pain intensity. Both were characterised by low Cronbach's alpha's. To the best of our knowledge no internationally validated standard scale is available to measure labour pain attitudes. In addition, the timing of the postnatal questionnaire may influence the findings. We have chosen a 2-weeks timeframe to avoid problems of inaccurate recall of labour pain intensity [77]. It is unlikely that this timing undermines the measurement of personal control in pain relief and the self-reported use of pain medication during labour since previous research has shown that respondents displayed a very good memory for the context of labour pain (e.g. pain management) [78]. However, because of this timeframe, feelings of relief, happiness or excitement as a reaction to the birth of a child - in the literature referred to as the 'halo effect' [79] - may bias the reported personal control in pain relief. Positive birth experiences may result in an overestimation of personal control, while a traumatic birth experience may yield an underestimation.

Third, the main goal of the broader study and data collection was to compare women's expectations and experiences with home and hospital births in Belgium and the Netherlands. For this paper we excluded women who actually had a home birth, because pharmacological pain relief is restricted to the hospital. Those planning for a home birth who are referred to the hospital are however included in our analysis. In the Netherlands this group was more likely than women with a planned hospital birth, to report the use of labour pain medication.

Fourth, our model is far from complete: other factors have proved to be determining the use of pain medication during labour. For example, Hodnett [80,81] found that continuous support of care providers reduced the likelihood of pain relief.

The merits of this research lie in the cross-national comparison and the longitudinal design. The introduction of care context allowed us to address the relative impact of antenatal pain acceptance and personal control in pain relief in two models of maternity care. Our findings illustrate that the childbirth context interferes with individual women's pain acceptance and personal control in pain relief with regard to the prediction of pain medication use. The repeated measurement design of this investigation contributes to the validity of our findings in terms of causality.

## Conclusion

Two main findings emerge from this investigation. First of all, the care context is of major importance when studying the use of pain medication during labour. This is illustrated by the fact that the answer to our first research question - do labour pain acceptance and personal control in pain relief determine how childbearing women cope with labour pain? - is country specific. Regarding Dutch women we find that the use of pain medication is lowest if women have a positive attitude towards labour pain and experience control over the reception of pain medication. In the Netherlands Gomar and Fernandez' [7] argument that the accessibility of, or control over, pain medication is likely to be one of the best predictors of the use of pain medication if women have negative attitudes towards labour pain, is confirmed by our findings. Pain medication use in Belgium hospital maternity care is high and very sensitive to negative attitudes towards labour pain. Even in women who report little personal control, hence much professional control, in pain relief, pain acceptance reduces the likelihood of pain medication use. This finding suggests that the Belgian obstetricians and midwives take the labour pain attitudes of childbearing women into account when deciding on pain medication use. In conclusion, while personal control in pain relief is the main determinant of pain medication use in the Netherlands, labour pain acceptance is decisive in the labour pain medication use of Belgian women. This contradicts the hypotheses formulated in response to the second and third research question. We reasoned that care providers in a women-centred maternity care system, which is how the Dutch care context is described in the literature [46], would be more sensitive to childbearing women's labour pain preferences or attitudes, in comparison to the bio-medical oriented, more hospital-centred Belgian system. Our findings indicate the opposite: Belgian care providers seem to be more sensitive to women's requests for pain relief compared to Dutch care providers. Although in earlier Dutch research Van der Hulst et al. [82] concluded that women's preferences stimulate or inhibit the medicalisation of childbirth, with regard to the use of pharmacological pain relief this is in fact true mainly in Belgium. Thus, although pain acceptance is a personal attribute, the effectiveness of pain acceptance in the reduction of pain medication use depends on the care context.

Second, our investigation also indicates that the average labour pain acceptance is the same among our Belgian and Dutch respondents. Hence, a specific Dutch pain culture (as suggested by e.g., Senden [18] and DeVries [46]) does not seem to exist, at least not from the point of view of childbearing women. This finding suggests that we cannot characterise Belgian women as mainly approaching labour pain as a useless inconvenience and Dutch women as perceiving labour pain as serving a biological purpose. Since we are only able to draw on information about childbearing women, we cannot test whether the same finding accounts for Belgian and Dutch care providers' ideas about labour pain. In addition, this finding does not necessarily account for the whole Dutch population. It should be noted that there is some regional variation in the home birth rates in the Netherlands. However, Noord-Brabant, our sampling area, is likely to be a good representative of the Dutch maternity care model. In Noord-Brabant the number of deliveries under the care of a midwife (including both home and policlinical births) is rather high (78%) in comparison to the other Dutch provinces. Only Overijsel and Gelderland have more mid-wife led deliveries, 85% and 83%, respectively [54]. Since non-pharmacological management of labour pain is one of the specificities of a midwife-led birth, the attitude towards home births is likely to be correlated with pain attitudes. Thus, women from Noord-Brabant are likely to have rather positive home birth and pain attitudes and thus resemble the rest of the Netherlands more than the Belgian population, despite its closeness to the Belgian border.

Despite the fact that the Belgian and Dutch women in our sample share the same pain attitudes, the use of pain medication strongly differs between the groups. More Belgian (47.8%) than Dutch respondents (14.5%) receive pharmacological pain relief. This could be an indication of an unmet need among Dutch respondents. More Dutch women might have been disappointed with their hospital birth as a consequence. This could explain the earlier finding that Dutch women giving birth at hospital report lower childbirth satisfaction compared to Belgian women with a hospital birth [45].

Although our sample is not likely to be representative for the entire Belgian and Dutch population of women giving birth in hospital, our findings suggest implications for care providers and the organisation of maternity care. In Belgium, the use of pharmacological pain relief is high (66.6% in 2007) [53]. In order to reduce this level of use, attention should be paid to the development of positive pain attitudes in pregnant women. In the Netherlands a floor effect may be operating: perhaps pain acceptance is not influential in reducing pain relief in the Netherlands because pain medication use has already reached a minimal level. Still, non-acceptance does not lead to a high pain medication use either, which means that Dutch care providers should perhaps be more attentive to women's non-acceptance of labour pain in order to avoid disappointed mothers.

## Competing interests

The authors declare that they have no competing interests.

## Authors' contributions

WC designed the study, organised the data collection, formulated the research questions and was responsible for the analysis of the data. She drafted the manuscript and revised it; the manuscript was reported back to MV and PB. PB and WC contributed to the conception of the study. MV contributed to the analysis of the data. MV and PB critically reviewed draft versions of the manuscript. All authors contributed to the development of this manuscript. All authors read and approved the final manuscript.

## Pre-publication history

The pre-publication history for this paper can be accessed here:

http://www.biomedcentral.com/1472-6963/10/268/prepub
